# Determinants of Symptomatic Intracranial Hemorrhage After Endovascular Stroke Treatment: A Retrospective Cohort Study

**DOI:** 10.1161/STROKEAHA.121.036195

**Published:** 2022-06-08

**Authors:** Wouter van der Steen, Nadinda A.M. van der Ende, Katinka R. van Kranendonk, Vicky Chalos, Robert J. van Oostenbrugge, Wim H. van Zwam, Yvo B.W.E.M. Roos, Pieter J. van Doormaal, Adriaan C.G.M. van Es, Hester F. Lingsma, Charles B.L.M. Majoie, Aad van der Lugt, Diederik W.J. Dippel, Bob Roozenbeek

**Affiliations:** Department of Neurology (W.v.d.S., N.A.M.v.d.E., V.C., D.W.J.D., B.R.), Erasmus MC University Medical Center, Rotterdam, the Netherlands.; Department of Radiology and Nuclear Medicine (W.v.d.S., N.A.M.v.d.E., V.C., P.J.v.D., A.v.d.L., B.R.), Erasmus MC University Medical Center, Rotterdam, the Netherlands.; Department of Public Health (V.C., H.F.L.), Erasmus MC University Medical Center, Rotterdam, the Netherlands.; Department of Radiology and Nuclear Medicine (K.R.v.K., C.B.L.M.M.), Amsterdam University Medical Center, University of Amsterdam, the Netherlands.; Department of Neurology (Y.B.W.E.M.R.), Amsterdam University Medical Center, University of Amsterdam, the Netherlands.; Department of Neurology (R.J.v.O.), Maastricht University Medical Center, the Netherlands.; Department of Radiology and Nuclear Medicine (W.H.v.Z.), Maastricht University Medical Center, the Netherlands.; Department of Radiology, Leiden University Medical Center, the Netherlands (A.C.G.M.v.E.).

**Keywords:** adult, blood glucose, brain ischemia, humans, intracranial hemorrhages, retrospective studies

## Abstract

Symptomatic intracranial hemorrhage (sICH) is a serious complication after endovascular treatment for ischemic stroke. We aimed to identify determinants of its occurrence and location.

**Methods:**

We retrospectively analyzed data from the Dutch MR CLEAN trial (Multicenter Randomized Clinical Trial of Endovascular Treatment for Acute Ischemic Stroke in the Netherlands) and MR CLEAN registry. We included adult patients with a large vessel occlusion in the anterior circulation who underwent endovascular treatment within 6.5 hours of stroke onset. We used univariable and multivariable logistic regression analyses to identify determinants of overall sICH occurrence, sICH within infarcted brain tissue, and sICH outside infarcted brain tissue.

**Results:**

SICH occurred in 203 (6%) of 3313 included patients and was located within infarcted brain tissue in 50 (25%), outside infarcted brain tissue in 23 (11%), and both within and outside infarcted brain tissue in 116 (57%) patients. In 14 patients (7%), data on location were missing. Prior antiplatelet use, baseline systolic blood pressure, baseline plasma glucose levels, post-endovascular treatment modified treatment in cerebral ischemia score, and duration of procedure were associated with all outcome parameters. In addition, determinants of sICH within infarcted brain tissue included history of myocardial infarction (adjusted odds ratio, 1.65 [95% CI, 1.06–2.56]) and poor collateral score (adjusted odds ratio, 1.42 [95% CI, 1.02–1.95]), whereas determinants of sICH outside infarcted brain tissue included level of occlusion on computed tomography angiography (internal carotid artery or internal carotid artery terminus compared with M1: adjusted odds ratio, 1.79 [95% CI, 1.16–2.78]).

**Conclusions:**

Several factors, some potentially modifiable, are associated with sICH occurrence. Further studies should investigate whether modification of baseline systolic blood pressure or plasma glucose level could reduce the risk of sICH. In addition, determinants differ per location of sICH, supporting the hypothesis of varying underlying mechanisms.

**Registration:**

URL: https://www.isrctn.com/; Unique identifier: ISRCTN10888758.

Endovascular treatment (EVT) substantially improves the outcome of patients with ischemic stroke caused by an intracranial large vessel occlusion. Nevertheless, 3-month mortality (15%) and functional dependence (39%) remain high.^1^ For a large part, this can be attributed to postprocedural symptomatic intracranial hemorrhages (sICHs), which occur in ≈6% of patients treated with EVT.^2,3^ After sICH, the likelihood of poor functional outcome is increased 6-fold, and the 3-month mortality rate is increased to 66%.^4^

Better insight into the clinical, radiological, and treatment-related determinants of sICH may eventually aid in the development of clinical guidelines for the prediction and prevention of this devastating complication.^5,6^ In analyzing the problem, we decided it is important to differentiate sICHs based on their location.^7^ First, because the underlying mechanisms and determinants may vary according to the location. Second, because location carries prognostic value.^8–10^ Parenchymal hematomas have a poorer prognosis than hemorrhagic infarctions.^4,11,12^ The prognostic relevance of subarachnoid hemorrhage after EVT is unsure.^10,13^

Prior studies investigating the determinants of sICH occurrence and location were small and had widely varying definitions of sICH.^5^ Therefore, robust evidence is limited. The aim of this study was to evaluate the association of clinical, radiological, and treatment-related characteristics with the occurrence and location of sICH in a large cohort of patients with anterior circulation ischemic stroke treated with EVT.

## Methods

### Study Design and Patients

We retrospectively analyzed data from MR CLEAN (Multicenter Randomized Clinical Trial of Endovascular Treatment for Acute Ischemic Stroke in the Netherlands) and the MR CLEAN registry. The MR CLEAN trial was a phase III multicenter clinical trial with randomized treatment group assignment, open-label treatment, and blinded outcome evaluation. EVT plus usual care (intervention group) was compared with usual care alone (control group). The MR CLEAN registry was a national, prospective, open, multicenter, observational monitoring study for stroke intervention centers that perform EVT in the Netherlands. It includes all patients with ischemic stroke who underwent EVT since the completion of the MR CLEAN trial in March 2014 until January 2019. We used data from patients registered before November 2017. Details on both the MR CLEAN trial and the MR CLEAN registry were published previously.^14,15^ The study protocols of the MR CLEAN trial and the MR CLEAN registry were both evaluated and approved by a central medical ethics committee and the research board of each participating center. The data of the MR CLEAN trial have been made publicly available at the Virtual International Stroke Trials Archive and can be accessed at http://www.virtualtrialsarchives.org/vista/. Individual patient data of the MR CLEAN registry cannot be made available under the Dutch law, as we did not obtain patient approval for sharing individual patient data, even in coded form. However, all syntax files and output of statistical analyses will be made available upon reasonable request.

For the current analysis, we included adult patients who underwent EVT (defined as entry into the angiography suite and receiving arterial puncture), were treated in a center that participated in the MR CLEAN trial, had a proximal intracranial large vessel occlusion in the anterior circulation (ie, internal carotid artery [ICA], internal carotid artery terminus, middle [M1/M2/M3] cerebral artery, or anterior [A1/A2] cerebral artery), and had an onset-to-groin puncture time of <6.5 hours. Alive patients with a follow-up of ≤1 day were considered to have an insufficient follow-up time to adequately determine sICH occurrence and were excluded from the analysis.

### Sources of Data and Measurement of Covariates

In both the MR CLEAN trial and the MR CLEAN registry, a trial office used case report forms to match patient data from electronic patients files with a specific study identification number. Using this number, all data were entered into a good clinical practice–approved, web-based clinical database (OpenClinica Community). The treating interventional radiologist provided data related to the endovascular procedure through a separate case report form. The study coordinators of the studies checked all data for completeness, formatting, and consistency. To check on reporting of safety, all discharge letters were screened for complications including sICH. For patients transferred from a referring stroke center to an intervention center, clinical and imaging data from the referring stroke center were collected and stored centrally. An imaging core laboratory assessed the images. The members of this core laboratory were blinded to all clinical findings, with the exception of clinical assessment of the occlusion location in case of baseline noncontrast computed tomography. For the MR CLEAN trial, observers were also blinded for treatment allocation (thrombectomy versus control). Before the assessment began, the observers were provided with guidelines including relevant definitions. In the MR CLEAN trial, the modified Thrombolysis in Cerebral Infarction was scored, while in the MR CLEAN registry, the extended Thrombolysis in Cerebral Infarction was scored. To be able to merge the data, we transformed the extended Thrombolysis in Cerebral Infarction of the MR CLEAN registry cohort to the modified Thrombolysis in Cerebral Infarction score by transforming grade 2C scores to grade 2B scores.

### Outcomes

In both the MR CLEAN trial and the MR CLEAN registry, sICH was defined as neurological deterioration (an increase of ≥4 points on the National Institutes of Health Stroke Scale [NIHSS]) and evidence of related ICH on follow-up imaging (noncontrast computed tomography or magnetic resonance imaging). Follow-up imaging was assessed by an imaging core laboratory that assessed ICH occurrence including location based on the Heidelberg Bleeding Classification.^7^ For statistical purposes, we dichotomized the location as sICH within infarcted brain tissue (sICH-WI) and sICH outside infarcted brain tissue (sICH-OI). Heidelberg Bleeding Classification class I or II hemorrhages (ie, hemorrhagic infarctions and parenchymal hematomas within or beyond infarcted brain tissue) were classified as sICH-WI, whereas Heidelberg Bleeding Classification class III hemorrhages (ie, parenchymal hematoma remote from infarcted brain tissue, intraventricular hemorrhage, subarachnoid hemorrhage, and subdural hemorrhage) were classified as sICH-OI. If neurological deterioration occurred but follow-up imaging could not be obtained by the research group, an serious adverse event committee assessed the discharge letter to decide whether or not sICH occurred.

### Statistical Analysis

We presented the baseline clinical, radiological, and treatment-related characteristics of the overall study population and stratified by occurrence of sICH. We presented medians and interquartile ranges or means and SDs for continuous variables and frequencies and percentages for categorical variables. We selected characteristics that we considered as potential determinants based on literature and expert opinion (Table S1). We used univariable and multivariable binary logistic regression models to evaluate the association of these characteristics with the overall occurrence of sICH (outcome 1), the occurrence of sICH-WI (outcome 2), and the occurrence of sICH-OI (outcome 3) within 90 days after intervention. Patients with both sICH-WI and sICH-OI were labeled as positive in both anatomic location outcomes (ie, outcomes 2 and 3).

For the multivariable models, we used a standard set of variables consisting of age, sex, baseline NIHSS score, baseline systolic blood pressure (SBP), baseline glucose level, Alberta Stroke Program Early CT Score on noncontrast computed tomography, computed tomography angiography collateral score, treatment with intravenous thrombolysis (IVT), performed endovascular procedure, post-EVT modified treatment in cerebral ischemia (mTICI) score, time from onset to groin puncture, and duration of endovascular procedure. In addition, we included factors with a *P* of <0.10 in univariable analysis. Results were presented as adjusted odds ratios with 95% CIs.

All statistical analyses were performed using R, version 4.0.5 (www.cran.r-project.org), with the packages Hmisc, rms, tableone, and dplyr. For univariable and multivariable regression analyses, we replaced missing values with multiple imputation (n=5 imputation sets) using the aregImpute function. More details regarding the multiple imputation approach are given in the Supplemental Material. Continuous factors were assessed for nonlinearity with restricted cubic spline functions with 3 knots. When a univariable nonlinear relationship was found between a continuous factor and outcome measure, we compared model fit of the multivariable regression model with a linear function to multivariable regression model with a nonlinear function.

## Results

### Patients

**Figure 1.**
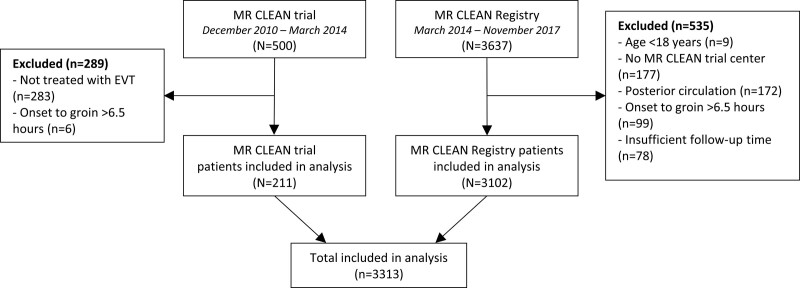
**Flowchart of patients included for the analysis.** EVT indicates endovascular treatment; and MR CLEAN, Multicenter Randomized Clinical Trial of Endovascular Treatment for Acute Ischemic Stroke in the Netherlands.

Five hundred patients were included in the MR CLEAN trial, and 3637 patients were included in the MR CLEAN registry between March 2014 and November 2017. For this analysis, we excluded 289 patients of the MR CLEAN trial who did not undergo EVT (n=283) or had an onset-to-groin puncture time of >6.5 hours (n=6; Figure 1). We excluded 535 patients of the MR CLEAN registry who were aged under 18 years (n=9), had no treatment in an MR CLEAN trial center (n=177), had an intracranial occlusion of the posterior circulation (n=172), had an onset-to-groin puncture time of >6.5 hours (n=99), or had insufficient follow-up time to adequately determine sICH occurrence (n=78). In total, 3313 patients were available for the analysis.

### Patient Characteristics

Median age was 72 years, 1735 (52%) patients were men, and the median baseline NIHSS was 16 (Table 1). Most patients had an M1 occlusion (58%), followed by an ICA or ICA-terminus occlusion (26%), and an M2 occlusion (14%). The median Alberta Stroke Program Early CT Score was 9. The majority of included patients received IVT before EVT (77%). Median onset-to-groin puncture time was 196 minutes, and median duration of procedure was 60 minutes. In total, 4.2% (5789/139 146) of the data points of the evaluated patient characteristics were missing.

**Table 1.**
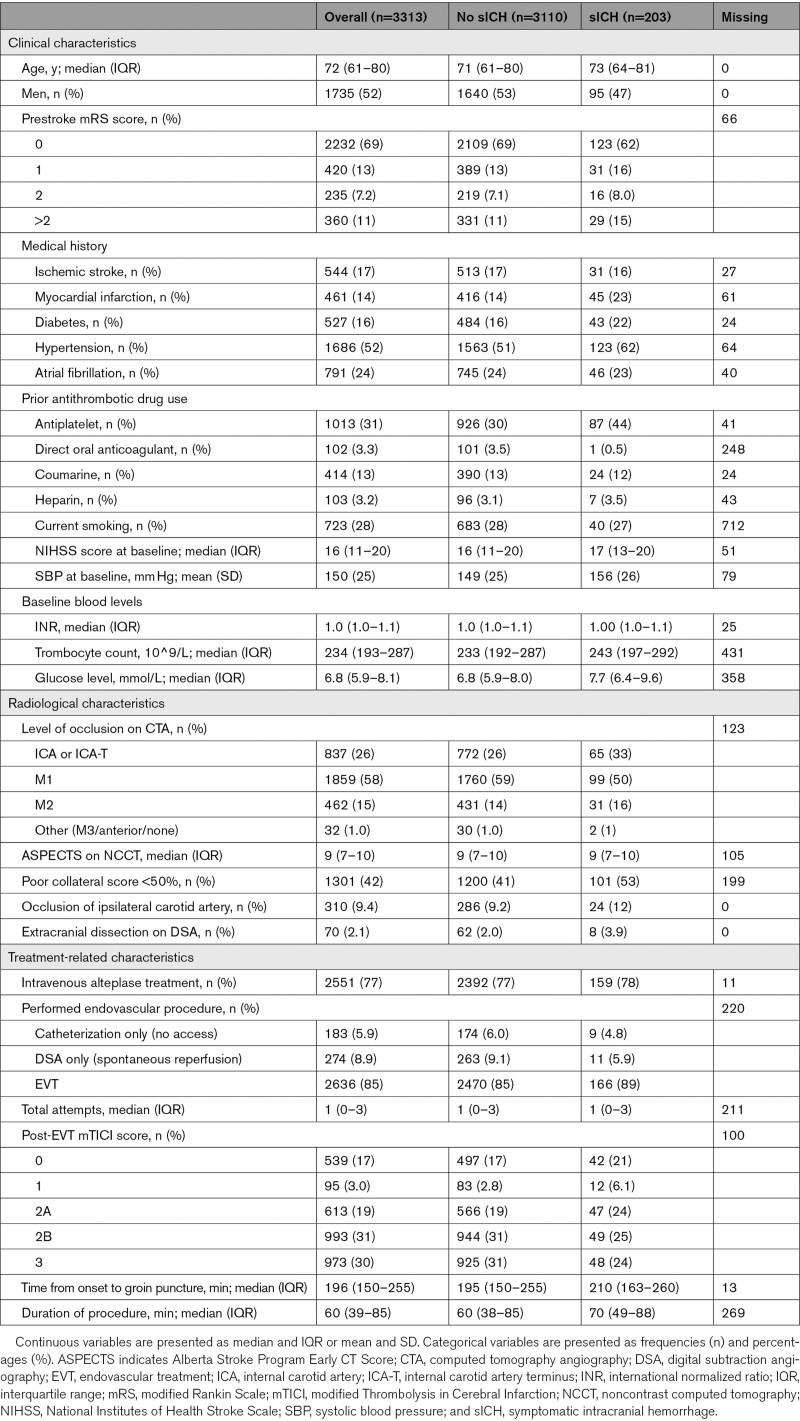
Baseline Characteristics of the Study Population, Overall and Stratified by the Occurrence of sICH

### Outcomes

SICH occurred in 203 (6%) patients. Of these, 50 (25%) were located within infarcted brain tissue, 23 (11%) outside infarcted brain tissue, and 116 (57%) both within and outside infarcted brain tissue (Table 2). In 14 patients (7%), data on location were missing.

**Table 2.**
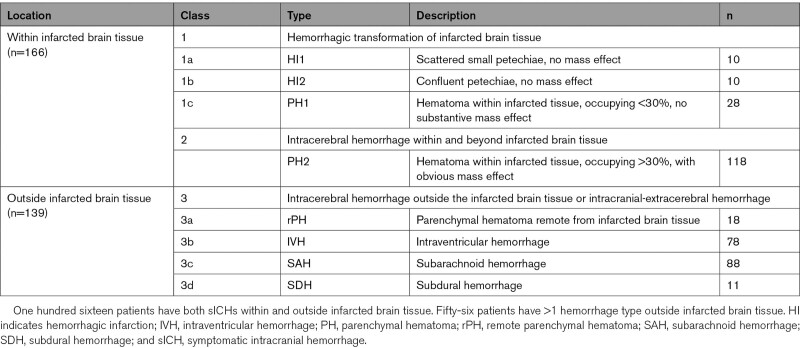
sICHs Classified According to the Heidelberg Bleeding Classification and Dichotomized by Location (Within or Outside Infarcted Brain Tissue)

### Nonlinear Associations

Baseline blood glucose level and duration of procedure had a nonlinear relationship with overall sICH occurrence, sICH-WI, and sICH-OI (Figure S1). Model fit of multivariable regression models with restricted cubic splines for glucose level and duration of procedure also showed a better model fit than the same models without nonlinear functions (likelihood-ratio test: *P*≤0.001 for sICH occurrence, *P*≤0.001 for sICH-WI, and *P*=0.001 for sICH-OI).

### Determinants of sICH Occurrence

**Figure 2.**
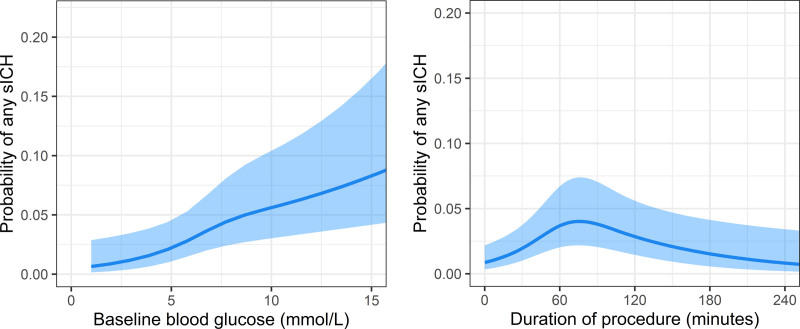
**Relationship of baseline blood glucose level and duration of procedure with overall symptomatic intracranial hemorrhage (sICH) occurrence.** The model is fitted with a restricted cubic spline function with 3 knots for baseline blood glucose level and duration of procedure. Lines represent the probability of any sICH with its 95% CI (light blue area) for a typical patient (a male patient, with a medical history of hypertension, prior treatment with intravenous alteplase, and median values for continuous factors). Both variables showed similar relationships to sICH within infarcted brain tissue and sICH outside infarcted brain tissue.

In multivariable analysis, history of myocardial infarction, prior antiplatelet use, increased baseline SBP, increased baseline glucose levels, poor collateral score, and extracranial dissection were associated with overall sICH occurrence (Table 3). Duration of procedure showed an inversed U-shaped relationship with sICH occurrence with the inflection point at ≈77 minutes (Figure 2). Compared with patients with post-EVT mTICI score 3, patients with post-EVT mTICI score 1 had the highest risk of sICH, followed by post-EVT mTICI score 0 and 2A. In addition, patients with an ICA or ICA-terminus occlusion had an increased risk of sICH compared with patients with an M1 occlusion. Age, history of diabetes or hypertension, prior antihypertensive drug use, NIHSS at baseline, and onset-to-groin puncture time only showed an association in univariable analysis (Table S2). Among others, history of atrial fibrillation, prior coumarin or DOAC use, Alberta Stroke Program Early CT Score, bridging IVT, and stent placement in ICA were not associated with sICH occurrence.

**Table 3.**
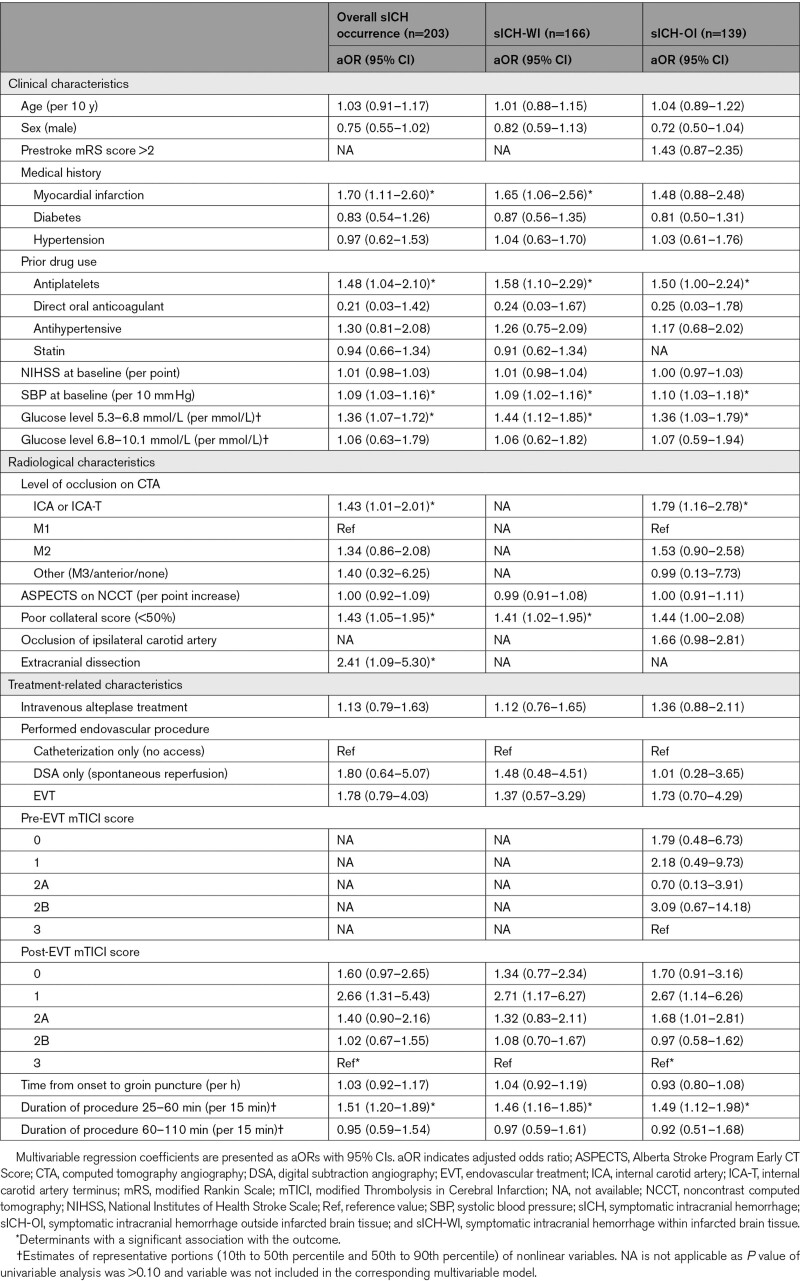
Multivariable Regression Analysis of Determinants of sICH Occurrence, sICH-WI and sICH-OI

### Determinants of sICH-WI

Most determinants of sICH-WI corresponded with the determinants of overall sICH occurrence (Table 3). However, this did not apply for extracranial dissection and level of occlusion, which both had a *P* of >0.10 in univariable analysis, and was not included in the multivariable model (Table S2).

### Determinants of sICH-OI

As opposed to overall sICH and sICH-WI, prestroke mRS score, pre-EVT mTICI score, and occlusion of ipsilateral carotid artery had a *P* of <0.10 in univariable analysis for sICH-OI and were included in the multivariable model (Table S2). Of the determinants of overall sICH occurrence, history of myocardial infarction, poor collateral score, and extracranial dissection were not associated with sICH-OI (Table 3).

## Discussion

In this large retrospective study of 2 combined databases, we found several clinical, radiological, and treatment-related determinants, which are associated with sICH occurrence after EVT for anterior large vessel occlusion strokes. In addition, we found that history of myocardial infarction and poor collateral score are associated with sICH-WI, whereas level of occlusion is associated with sICH-OI.

### Determinants of sICH Occurrence

Most determinants of sICH observed in our study correspond to risk factors found in previous studies.^5^ This includes potentially modifiable factors, such as SBP and plasma glucose level at baseline. Whether modification of these factors would reduce the risk of sICH occurrence after EVT should be investigated in randomized controlled trials. Results of the BP-TARGET trial (Blood Pressure Target in Acute Stroke to Reduce Hemorrhage After Endovascular Therapy; https://www.clinicaltrials.gov; unique identifier: NCT03160677) showed no effect of blood pressure reduction on hemorrhage occurrence after EVT.^16^ However, this might be attributed to the moderate difference in mean hourly SBP during the first 24 hours between the intervention and control arms of the trial. Therefore, results of ongoing randomized trials, including ENCHANTED-2 (Enhanced Control of Hypertension and Thrombolysis Stroke Study-2; https://www.clinicaltrials.gov; unique identifier: NCT04140110), which investigates a more stringent SBP control, are needed to give more clarity on this issue.

Our study was the first to find that extracranial dissections are associated with a higher sICH risk after EVT. Possible explanations for this finding may be that extracranial dissections have a higher risk of giving tandem occlusions and a lack of collateral circulation.^17–19^ However, since our study was the first to find this association, it should be evaluated further in other studies.

Remarkably, we did not find any associations with markers of baseline infarct size (ie, baseline NIHSS score and Alberta Stroke Program Early CT Score), even though these factors were found to be associated with sICH occurrence in various previous studies.^5,9,20–22^ This might be partly explained by the differences in study populations, variation in coding of determinants (continuous versus dichotomous), or different definitions of sICH. It is important to find out what the true association between baseline infarct size and sICH is and what markers of infarct size could best be used to predict sICH. Therefore, further research is needed with computed tomography perfusion and magnetic resonance imaging as imaging techniques to assess baseline infarct size.^23,24^

Furthermore, we also found no association with bridging IVT and sICH occurrence. This is in accordance with the results of recently published randomized controlled trials (DIRECT-MT [Direct Intraarterial Thrombectomy in Order to Revascularize Acute Ischemic Stroke Patients With Large Vessel Occlusion Efficiently in Chinese Tertiary Hospitals: A Multicenter Randomized Clinical Trial)], DEVT [Direct Endovascular Treatment], and MR CLEAN-NOIV [A Randomized Trial of Intravenous Alteplase Before Endovascular Treatment for Stroke]) evaluating the efficacy and safety of bridging IVT before EVT.^25–28^ All trials found no statistically significant difference in sICH occurrence between EVT alone or bridging IVT. However, as this could very well be due to the low rate of sICH occurrence and subsequent lack of power in the separate trials, we expect that pooled results, including results of ongoing trials (SWIFT DIRECT [Solitaire With the Intention for Thrombectomy Plus Intravenous t-PA Versus DIRECT Solitaire Stent-Retriever Thrombectomy in Acute Anterior Circulation Stroke], and DIRECT-SAFE [A Randomized Controlled Trial of DIRECT Endovascular Clot Retrieval Versus Standard Bridging Thrombolysis With Endovascular Clot Retrieval]), will provide more clarity concerning this issue.

Lastly, we found that patients with poor reperfusion scores had an increased risk of sICH occurrence. This is in accordance with earlier findings and is probably the result of the higher follow-up infarct volume at risk of bleeding.^5,21^ Interestingly, we also found that patients with limited reperfusion are at higher risk than patients with no reperfusion. This could well be associated to the widespread concept of reperfusion injury.^29^

### Determinants of sICH-WI

We found that history of myocardial infarction and poor collateral score were associated with sICH-WI, while these factors were not associated with sICH-OI. History of myocardial infarction may be associated with sICH-WI, due to the often concomitant generalized atherosclerotic vasculopathy.^30^ In addition, history of myocardial infarction increases the risk of cardioembolic strokes, which have also been associated with hemorrhagic transformation of infarcted brain tissue.^31,32^ Then again, we did not find an association with history of atrial fibrillation, which is the main cause of cardioembolic strokes.^31^ This finding contradicts earlier results and should, therefore, be evaluated further.^11,12^ Prior research showed that poor collateral scores are associated with parenchymal hematomas, which include most sICH-WI.^22^ This might be explained by the higher degree of hypoperfusion of brain tissue and concomitant infarct growth.^33^

### Determinants of sICH-OI

Relatively few studies have investigated determinants of sICH-OI. If so, these studies investigated determinants of subarachnoid hemorrhage.^10,13^ Although the number of events in these studies is low, the consensus is that mainly procedural complications are associated with subarachnoid hemorrhage.^2,9^ We did not find an association with treatment-related characteristics such as performed endovascular procedure, total attempts, and stent placement in ICA. However, we did find an association with post-EVT mTICI score and duration of procedure. The latter is partly in accordance with an earlier study showing a higher risk of sICH and complications with increasing procedure times.^34^ However, this prior study showed an exponential growth curve, whereas we found an inversed U-shaped relationship. This difference might be caused by the low number of cases and limited statistical power of this nonlinear analysis. The true relationship should be further evaluated in future larger studies. Lastly, we found that the level of occlusion was associated with sICH-OI but not with sICH-WI. This mainly concerned a higher risk for ICA or ICA-terminus occlusions. The underlying mechanism behind this association should be further investigated but may be associated to the added risk of cerebral hyperperfusion syndrome after spontaneous or mechanical carotid revascularization.^19^

### Limitations

Our study has limitations. First, this was an explorative study, in which we analyzed many parameters. Automatically, this comes with a higher risk of type 1 errors due to multiple testing. Therefore, the results should be interpreted with caution and should be reevaluated in other cohorts. Second, not all patients in the MR CLEAN registry had follow-up imaging. Therefore, we cannot rule out the possibility of missing sICH in this group. However, this uncertainty is limited since it is standard procedure in the Netherlands to perform a computed tomography scan in case of neurological deterioration. Third, although we had access to a large cohort of consecutive patients, the occurrence of sICH was relatively low. This mainly concerned the subanalysis for determinants of sICH-OI. To increase statistical power in this analysis, we aggregated the different subtypes of sICH-OI in one group. However, we cannot rule out that the determinants of the underlying subtypes vary. Lastly, of patients with sICH, a relatively large group had a combined intracranial hemorrhage within and outside infarcted brain tissue. For these patients, it was not known which of the hemorrhages was symptomatic. Hence, we labeled them as positive in both location groups, reducing variability. It is interesting to see that despite this conservative approach, we still found differences between both groups.

### Conclusions

Several factors, including potentially modifiable factors, that is, baseline SBP and glucose level, are associated with sICH occurrence. Whether modification of these factors will reduce the risk of sICH needs to be evaluated. In addition, determinants differ per location of sICH. Further research should evaluate which underlying mechanisms influence this location.

## Article Information

### Acknowledgments

We thank the MR CLEAN trial (Multicenter Randomized Clinical Trial of Endovascular Treatment for Acute Ischemic Stroke in the Netherlands) and MR CLEAN registry investigators for their contribution.

### Sources of Funding

The MR CLEAN trial (Multicenter Randomized Clinical Trial of Endovascular Treatment for Acute Ischemic Stroke in the Netherlands) was supported by the Dutch Heart Foundation and by unrestricted grants from AngioCare Covidien/ev3, Medac/Lamepro, Stryker, and Penumbra. The MR CLEAN registry was partly funded by the TWIN Foundation and by the Erasmus MC University Medical Center, Maastricht UMC, and Amsterdam UMC.

### Disclosures

Drs Dippel and van der Lugt report funding from the Dutch Heart Foundation, Brain Foundation Netherlands, The Netherlands Organisation for Health Research and Development, Health Holland Top Sector Life Sciences and Health, and unrestricted grants from Penumbra, Inc, Stryker, Medtronic, Thrombolytic Science, LLC, and Cerenovus for research, all paid to institution. Dr Majoie received funds from the TWIN Foundation (related to this project, paid to institution) and from CVON/Dutch Heart Foundation, Stryker, European Commission, and Health Evaluation Netherlands (unrelated; all paid to institution). Drs Majoie and Roos are minor shareholders of Nico.lab—a company that focuses on the use of artificial intelligence for medical imaging analysis. Dr van Zwam reports speaker fees from Stryker and Cerenovus, both paid to the institution, compensation from Philips for data and safety monitoring services, and grants from Johnson and Johnson International. Dr van Doormaal reports compensation from Stryker for consultant services.

### Supplemental Material

Supplemental Methods

Tables S1 and S2

Figure S1

MR CLEAN Trial and MR CLEAN Registry Investigators

## Supplementary Material


