# Supplementary material for: Determinants of Symptomatic Intracranial Hemorrhage After Endovascular Stroke Treatment: A Retrospective Cohort Study
Source: Stroke. 2022 Jun 8;53(9):2818–27. doi: 10.1161/STROKEAHA.121.036195 (PMC9389940; doi:10.1161/STROKEAHA.121.036195)
Supplement: Supplementary file 1 [file str-53-2818-s001.pdf]

SUPPLEMENTAL MATERIAL.

DETERMINANTS OF SYMPTOMATIC INTRACRANIAL HEMORRHAGE AFTER ENDOVASCULAR STROKE TREATMENT: A RETROSPECTIVE COHORT STUDY

Wouter van der Steen, MD[1,2]; Nadinda A.M. van der Ende, MD[1,2]; Katinka R. van Kranendonk, MD[3]; Vicky Chalos, MD[1,2,4]; Robert J. van Oostenbrugge, MD, PhD[5]; Wim van Zwam, MD, PhD[6]; Yvo B.W.E.M. Roos, MD, PhD[7]; Pieter J. van Doormaal, MD[1]; Adriaan C.G.M. van Es, MD, PhD[8]; Hester F. Lingsma, PhD[4]; Charles B.L.M. Majoie, MD, PhD[3]; Aad van der Lugt, MD, PhD[2]; Diederik W.J. Dippel, MD, PhD[1]; Bob Roozenbeek, MD, PhD[1,2]; on behalf of the MR CLEAN trial and MR CLEAN Registry investigators

Affiliations:

1. Department of Neurology, Erasmus MC University Medical Center, Rotterdam, The Netherlands
2. Department of Radiology & Nuclear Medicine, Erasmus MC University Medical Center, Rotterdam, The Netherlands
3. Department of Radiology & Nuclear Medicine, Amsterdam University Medical Center, University of Amsterdam, Amsterdam, The Netherlands
4. Department of Public Health, Erasmus MC University Medical Center, Rotterdam, The Netherlands
5. Department of Neurology, Maastricht University Medical Center, Maastricht, The Netherlands
6. Department of Radiology & Nuclear Medicine, Maastricht University Medical Center, Maastricht, The Netherlands
7. Department of Neurology, Amsterdam University Medical Centers, University of Amsterdam, Amsterdam, The Netherlands
8. Department of Radiology, Leiden University Medical Center, Leiden, The Netherlands

Table of Contents

Supplemental Methods……………………………………………………………….. 3
Multiple Imputation Model……………………………………………………….. 3
Supplemental Tables……………………………………………………………….. 4
Table I………………………………………………………………………….. 4
Table II…………………………………………………………………………. 5
Supplemental Figure……………………………………………………………….. 7
Figure I…………………………………………………………………………. 7
Supplemental Investigator List……………………………………………………... 8
MR CLEAN Registry Investigators……………………………………………….. 8
MR CLEAN Trial Investigators…………………………………………………… 9
List of affiliations………………………………………………………………….. 10

Supplemental Methods

Multiple imputation model

For univariable and multivariable regression analyses, we replaced missing values with multiple imputation using the aregImpute function of the Hmisc package in R version 4.0.5 (www.cran.r-project.org). In the model we included all the variables that were considered as potential determinants (Table I). In addition, we added the outcomes sICH occurrence, sICH-WI, and sICH-OI. There were no missings in sICH occurrence, however, in 14 patients we imputed missing sICH locations. The reason for these missing locations are related to technical issues (missing NCCT scan), and there is no indication that this is associated with the location of the hemorrhage. We performed 5 multiple imputation sets, in which we used 3 knots for continuous variables.

Supplemental Tables

**Table I.** Baseline clinical, radiological and treatment-related variables that we considered as potential determinants.	
**Clinical characteristics**	**Radiological characteristics**
Age	ASPECTS on NCCT
Sex	Nucleus lentiformis ischemia
Pre-stroke mRS score	Level of occlusion on CTA
Medical history	Dense artery sign
Ischemic stroke	Poor collateral score <50%
Ischemic stroke in same vascular area	Stenosis of ipsilateral carotid artery
Myocardial infarction	Occlusion of ipsilateral carotid artery
Peripheral artery disease	
Diabetes Mellitus	Extracranial dissection on DSA
Hypertension	
Atrial fibrillation	**Treatment-related characteristics**
Hypercholesterolemia	
Prior drug use	Intravenous alteplase treatment
Antiplatelet	Performed endovascular procedure*
Direct oral anticoagulant	
Coumarine	Total attempts
Heparin	Use of balloon guided catheter
Antihypertensive	Periprocedural use of heparin
Statin	Stent placement in ICA
Current smoking	Pre-EVT mTICI score
NIHSS score at baseline	Post-EVT mTICI score
SBP at baseline	Time from onset to groin puncture
Baseline blood levels	
INR	Duration of procedure
Trombocyte level	
Glucose level	
Creatinine level	

mRS indicates modified Ranking Scale; INR, International Normalized ratio; NIHSS, National Institute of Health Stroke Scale; SBP, systolic blood pressure; ASPECTS, Alberta Stroke Program Early CT score; NCCT, Non-Contrast CT; CTA, CT Angiography; DSA, Digital Substraction Angiography; EVT, Endovascular Treatment; mTICI, modified Thrombolysis in Cerebral Infarction.
**Catheterization only, DSA only or Endovascular Treatment.*

**Table II.** Univariable regression analysis of determinants of overall sICH occurrence, sICH within infarcted brain tissue and sICH outside infarcted brain tissue			
**Determinants**	**overall sICH occurrence (n=203)**	**sICH within infarcted brain tissue (n=166)**	**sICH outside infarcted brain tissue (n=139)**
**Clinical characteristics**	**OR (95% CI)**	**OR (95% CI)**	**OR (95% CI)**
Age (per 10 years)	1.16 (1.04-1.29)*	1.16 (1.03-1.30)*	1.19 (1.05-1.35)*
Sex (male)	0.79 (0.59-1.05)	0.84 (0.62-1.14)	0.77 (0.55-1.07)
Pre-stroke mRS score > 2	1.39 (0.92-2.09)	1.43 (0.93-2.21)	1.52 (0.96-2.40)*
Medical history			
Ischemic stroke	0.91 (0.61-1.35)	0.88 (0.58-1.35)	0.85 (0.53-1.36)
Ischemic stroke in same vascular area	1.19 (0.70-2.03)	1.12 (0.62-2.03)	1.13 (0.56-2.30)
Myocardial infarction	1.92 (1.34-2.74)*	2.00 (1.38-2.91)*	1.69 (1.10-2.59)*
Peripheral artery disease	1.00 (0.61-1.66)	1.07 (0.63-1.81)	0.89 (0.48-1.65)
Diabetes Mellitus	1.46 (1.03-2.07)*	1.61 (1.12-2.32)*	1.48 (0.99-2.23)*
Hypertension	1.53 (1.14-2.06)*	1.64 (1.19-2.26)*	1.58 (1.11-2.25)*
Atrial fibrillation	0.94 (0.67-1.32)	0.94 (0.65-1.35)	0.84 (0.56-1.26)
Hypercholesterolemia	1.14 (0.84-1.54)	1.22 (0.88-1.68)	1.06 (0.74-1.52)
Prior drug use			
Antiplatelets	1.79 (1.33-2.39)*	1.96 (1.44-2.66)*	1.80 (1.28-2.53)*
Direct oral anticoagulant	0.25 (0.05-1.31)*	0.28 (0.05-1.51)*	0.26 (0.04-1.85)*
Coumarine	0.93 (0.60-1.44)	0.82 (0.50-1.35)	0.71 (0.40-1.26)
Heparin	1.14 (0.52-2.49)	0.88 (0.36-2.20)	1.36 (0.58-3.16)
Antihypertensive	1.61 (1.20-2.17)*	1.69 (1.22-2.33)*	1.57 (1.10-2.23)*
Statin	1.33 (0.99-1.77)*	1.40 (1.02-1.91)*	1.33 (0.94-1.87)
Current smoking	1.03 (0.71-1.50)	1.02 (0.67-1.55)	1.11 (0.70-1.75)
NIHSS at baseline (per point)	1.03 (1.01-1.05)*	1.03 (1.01-1.06)*	1.02 (1.00-1.05)*
SBP at baseline (per 10 mmHg)	1.12 (1.06-1.18)*	1.12 (1.06-1.19)*	1.14 (1.07-1.21)*
Baseline blood levels			
INR (per value)	0.89 (0.61-1.29)	0.91 (0.61-1.34)	0.77 (0.47-1.28)
Trombocyte count (per 10 * 10^9/L)	1.01 (0.99-1.02)	1.01 (0.99-1.02)	1.01 (0.99-1.03)
Creatinine level (per 10 µmol/L)	0.99 (0.95-1.04)	1.00 (0.96-1.04)	1.00 (0.96-1.05)
**Radiological characteristics**			
Level of occlusion on CTA			
ICA or ICA-T	1.53 (1.11-2.11)	1.44 (1.02-2.02)	2.06 (1.39-3.04)
M1	Ref.*	Ref.	Ref.*
M2	1.26 (0.83-1.90)	1.08 (0.68-1.70)	1.46 (0.89-2.41)
Other (M3/anterior/none)	1.16 (0.27-4.90)	1.27 (0.30-5.41)	0.88 (0.12-6.59)
ASPECTS on NCCT (per point increase)	0.97 (0.90-1.05)	0.97 (0.89-1.05)	0.98 (0.90-1.08)
Nucleus lentiformis ischemia	0.87 (0.63-1.19)	0.85 (0.61-1.21)	0.91 (0.63-1.32)
Dense artery sign	1.17 (0.85-1.61)	1.25 (0.90-1.74)	1.10 (0.76-1.58)
Poor collateral score (<50%)	1.63 (1.22-2.16)*	1.62 (1.19-2.20)*	1.65 (1.18-2.32)*
Stenosis of ipsilateral carotid artery	1.24 (0.77-2.01)	1.08 (0.62-1.88)	1.10 (0.60-2.00)
Occlusion of ipsilateral carotid artery	1.32 (0.85-2.06)	1.36 (0.85-2.19)	1.72 (1.07-2.75)*
Extracranial dissection	2.02 (0.95-4.27)*	1.93 (0.85-4.38)	1.88 (0.75-4.69)
**Treatment-related characteristics**			
Intravenous alteplase treatment	1.07 (0.76-1.50)	1.06 (0.73-1.53)	1.23 (0.81-1.88)
Performed endovascular procedure			
Catheterization only (no access)	Ref.	Ref.	Ref.*
DSA only (spontaneous reperfusion)	0.85 (0.33-2.16)	0.83 (0.30-2.29)	0.51 (0.19-1.39)
Endovascular treatment	1.29 (0.66-2.54)	1.18 (0.58-2.40)	1.14 (0.55-2.34)
Total attempts (per extra attempt)	1.01 (0.93-1.09)	1.01 (0.93-1.10)	1.02 (0.93-1.12)

Use of balloon guided catheter	0.85 (0.63-1.15)	0.85 (0.61-1.18)	0.96 (0.68-1.36)
Periprocedural use of heparin	1.22 (0.88-1.69)	1.21 (0.86-1.72)	1.20 (0.83-1.74)
Stent placement in ICA	1.23 (0.72-2.12)	1.33 (0.76-2.32)	0.90 (0.45-1.82)
Pre-EVT mTICI score			
0	1.25 (0.62-2.51)	1.25 (0.59-2.64)	2.14 (0.79-5.80)
1	1.36 (0.56-3.28)	1.45 (0.57-3.67)	2.35 (0.72-7.67)
2A	0.90 (0.35-2.33)	0.91 (0.33-2.51)	0.69 (0.15-3.09)
2B	1.02 (0.41-2.52)	1.07 (0.40-2.87)	1.62 (0.48-5.45)
3	Ref.	Ref.	Ref.*
Post-EVT mTICI score			
0	1.60 (1.05-2.46)	1.43 (0.90-2.27)	1.77 (1.06-2.94)
1	2.70 (1.38-5.28)	2.75 (1.26-5.97)	2.87 (1.32-6.27)
2A	1.60 (1.06-2.42)	1.51 (0.97-2.36)	1.90 (1.18-3.06)
2B	1.04 (0.69-1.57)	1.11 (0.72-1.69)	1.10 (0.68-1.79)
3	Ref.*	Ref.*	Ref.*
Time from onset to groin puncture (per hr)	1.10 (0.98-1.23)	1.10 (0.98-1.25)	1.00 (0.87-1.14)

Univariable regression coefficients are presented as adjusted Odds Ratio (aOR) with 95% confidence interval (CI). Ref. indicates reference value; sICH indicates symptomatic Intracranial Hemorrhage; mRS, modified Rankin Scale; NIHSS, National Institutes of Health Stroke Scale; SBP, systolic blood pressure; INR, International Normalized Ratio; CTA, CT angiography; ICA(-T), internal carotid artery (terminus);M(segment), middle cerebral artery; ASPECTS, Alberta Stroke Program Early CT score; NCCT, Non-Contrast CT; DSA, Digital Subtraction Angiography; EVT, Endovascular Treatment; mTICI, modified Thrombolysis in Cerebral Infarction.

**Odds Ratios with a p-value < 0.10*

Supplemental Figure

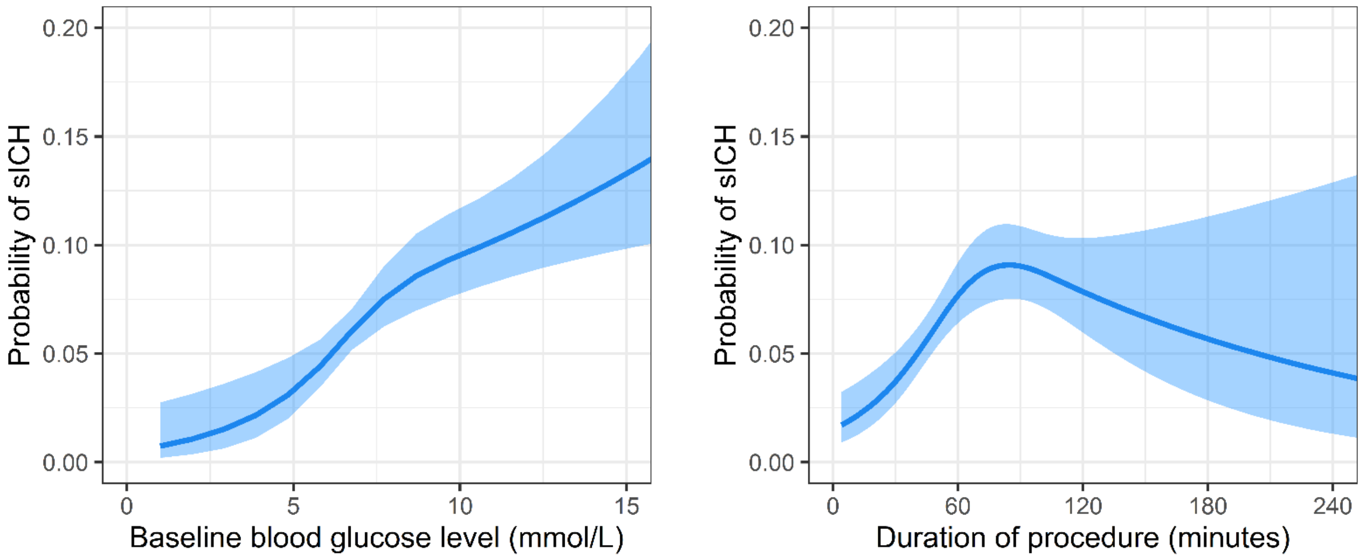

Figure I. Non-linear univariable relationship of baseline blood glucose level (left figure) and duration of procedure (right figure) with overall sICH occurrence (Blue lines). Lightblue areas indicate 95% confidence interval. Both variables showed similar non-linear univariable relationships to sICH occurrence within infarcted brain tissue and sICH occurrence outside infarcted brain tissue.

Supplemental Investigator List

Investigator list and affiliations of MR CLEAN Registry and MR CLEAN Trial Investigators

MR CLEAN Registry Investigators

Executive committee

Diederik W.J. Dippel[1];Aad van der Lugt[2];Charles B.L.M. Majoie[3];Yvo B.W.E.M. Roos[4];Robert J. van Oostenbrugge[5];Wim H. van Zwam[6];Jelis Boiten[14];Jan Albert Vos[8]

Study coordinators

Ivo G.H. Jansen[3];Maxim J.H.L. Mulder[1,2];Robert- Jan B. Goldhoorn[5,6];Kars C.J. Compagne[2];Manon Kappelhof[3];Josje Brouwer[4];Sanne J. den Hartog[1,2,40];Wouter H. Hinsenveld [5,6];

Local principal investigators

Diederik W.J. Dippel[1];Bob Roozenbeek[1];Aad van der Lugt[2];Adriaan C.G.M. van Es[2];Charles B.L.M. Majoie[3];Yvo B.W.E.M. Roos[4];Bart J. Emmer[3];Jonathan M. Coutinho[4];Wouter J. Schonewille[7];Jan Albert Vos[8]; Marieke J.H. Wermer[9];Marianne A.A. van Walderveen[10];Julie Staals[5];Robert J. van Oostenbrugge[5];Wim H. van Zwam[6];Jeannette Hofmeijer[11];Jasper M. Martens[12];Geert J. Lycklama à Nijeholt[13];Jelis Boiten[14];Sebastiaan F. de Bruijn[15];Lukas C. van Dijk[16];H. Bart van der Worp[17];Rob H. Lo[18];Ewoud J. van Dijk[19];Hieronymus D. Boogaarts[20];J. de Vries[22];Paul L.M. de Kort[21]; Julia van Tuijl[21] ; Jo P. Peluso[26];Puck Fransen[22];Jan S.P. van den Berg[22];Boudewijn A.A.M. van Hasselt[23];Leo A.M. Aerden[24];René J. Dallinga[25];Maarten Uyttenboogaart[28];Omid Eschgi[29];Reinoud P.H. Bokkers[29];Tobien H.C.M.L. Schreuder[30];Roel J.J. Heijboer[31];Koos Keizer[32];Lonneke S.F. Yo[33];Heleen M. den Hertog[22];Tomas Bulut[35]; Paul J.A.M. Brouwers[34]

Imaging assessment committee

Charles B.L.M. Majoie[3](chair);Wim H. van Zwam[6];Aad van der Lugt[2];Geert J. Lycklama à Nijeholt[13];Marianne A.A. van Walderveen[10];Marieke E.S. Sprengers[3];Sjoerd F.M. Jenniskens[27];René van den Berg[3];Albert J. Yoo[38];Ludo F.M. Beenen[3];Alida A. Postma[6];Stefan D. Roosendaal[3];Bas F.W. van der Kallen[13];Ido R. van den Wijngaard[13];Adriaan C.G.M. van Es[2];Bart J. Emmer[3];Jasper M. Martens[12]; Lonneke S.F. Yo[33];Jan Albert Vos[8]; Joost Bot[36], Pieter-Jan van Doormaal[2]; Anton Meijer[27];Elyas Ghariq[13]; Reinoud P.H. Bokkers[29];Marc P. van Proosdij[37];G. Menno Krietemeijer[33];Jo P. Peluso[26];Hieronymus D. Boogaarts[20];Rob Lo[18];Wouter Dinkelaar[2]Auke P.A. Appelman[29];Bas Hammer[16];Sjoert Pegge[27];Anouk van der Hoorn[29];Saman Vinke[20].

Writing committee

Diederik W.J. Dippel[1](chair);Aad van der Lugt[2];Charles B.L.M. Majoie[3];Yvo B.W.E.M. Roos[4];Robert J. van Oostenbrugge[5];Wim H. van Zwam[6];Geert J. Lycklama à Nijeholt[13];Jelis Boiten[14];Jan Albert Vos[8];Wouter J. Schonewille[7];Jeannette Hofmeijer[11];Jasper M. Martens[12];H. Bart van der Worp[17];Rob H. Lo[18]

Adverse event committee

Robert J. van Oostenbrugge[5](chair);Jeannette Hofmeijer[11];H. Zwenneke Flach[23]

Trial methodologist

Hester F. Lingsma[40]

Research nurses / local trial coordinators

Naziha el Ghannouti[1];Martin Sterrenberg[1];Wilma Pellikaan[7];Rita Sprengers[4];Marjan Elfrink[11];Michelle Simons[11];Marjolein Vossers[12];Joke de Meris[14];Tamara Vermeulen[14];Annet Geerlings[19];Gina van Vemde[22];Tiny Simons[30];Gert Messchendorp[28];Nynke Nicolaij[28];Hester Bongenaar[32];Karin Bodde[24];Sandra Kleijn[34];Jasmijn Lodico[34]; Hanneke Droste[34];Maureen Wollaert[5];Sabrina Verheesen[5];D. Jeurrissen[5];Erna Bos[9];Yvonne Drabbe[15];Michelle Sandiman[15];Nicoline Aaldering[11];Berber Zweedijk[17];Jocova Vervoort[21];Eva Ponjee[22];Sharon Romviel[19];Karin Kanselaar[19];Denn Barning[10].

PhD / Medical students

Esmee Venema[40]; Vicky Chalos[1,40]; Ralph R. Geuskens[3]; Tim van Straaten[19];Saliha Ergezen[1]; Roger R.M. Harmsma[1]; Daan Muijres[1]; Anouk de Jong[1];Olvert A. Berkhemer[1,3,6];Anna M.M. Boers[3,39]; J. Huguet[3];P.F.C. Groot[3];Marieke A. Mens[3];Katinka R. van Kranendonk[3];Kilian M. Treurniet[3];Manon L. Tolhuisen[3,39];Heitor Alves[3];Annick J. Weterings[3],Eleonora L.F. Kirkels[3],Eva J.H.F. Voogd[11];Lieve M. Schupp[3];Sabine L. Collette[28,29];Adrien E.D. Groot[4];Natalie E. LeCouffe[4];Praneeta R. Konduri[39];Haryadi Prasetya[39];Nerea Arrarte-Terreros[39];Lucas A. Ramos[39].

MR CLEAN Trial investigators

Olvert A. Berkhemer, M.D.,[*,1,3] Puck S.S. Fransen, M.D.,[*,1,2] Debbie Beumer, M.D.,[*1,5] Lucie A. van den Berg, M.D.,[4] Hester F. Lingsma, M.D., Ph.D.,[40] Albert J. Yoo, M.D.,[41] Wouter J. Schonewille, M.D.,[7] Jan Albert Vos, M.D., Ph.D.,[8] Paul J. Nederkoorn, M.D., Ph.D.,[4] Marieke J.H. Wermer, M.D., Ph.D.,[9] Marianne A.A. van Walderveen, M.D., Ph.D.,[10] Julie Staals, M.D., Ph.D.,[5] Jeannette Hofmeijer, M.D., Ph.D.,[11] Jacques A. van Oostayen, M.D., Ph.D.,[12] Geert J. Lycklama à Nijeholt, M.D., Ph.D.,[13] Jelis Boiten, M.D., Ph.D.,[14] Patrick A. Brouwer, M.D.,[2] Bart J. Emmer, M.D., Ph.D.,[2] Sebastiaan F. de Bruijn, M.D., Ph.D.,[15] Lukas C. van Dijk, M.D.,[16] L. Jaap Kappelle, M.D., Ph.D.,[17] Rob H. Lo, M.D.,[18] Ewoud J. van Dijk, M.D., Ph.D.,[19] Joost de Vries, M.D., Ph.D.,[20] Paul L.M. de Kort, M.D., Ph.D.,[21] Willem Jan J. van Rooij, M.D., Ph.D.,[26] Jan S.P. van den Berg, M.D., Ph.D.,[22] Boudewijn A.A.M. van Hasselt, M.D.,[23] Leo A.M. Aerden, M.D., Ph.D.,[24] René J. Dallinga, M.D.,[25] Marieke C. Visser, M.D., Ph.D.,[42] Joseph C.J. Bot, M.D., Ph.D.,[36] Patrick C. Vroomen, M.D., Ph.D.,[28] Omid Eshghi, M.D.,[29] Tobien H.C.M.L. Schreuder, M.D.,[30] Roel J.J. Heijboer, M.D.,[31] Koos Keizer, M.D., Ph.D.,[32] Alexander V. Tielbeek, M.D., Ph.D.,[33] Heleen M. den Hertog, M.D., Ph.D.,[34] Renske M. van den Berg-Vos, M.D., Ph.D.,[43] Giorgos B. Karas, M.D.,[44] Ewout W. Steyerberg, M.D., Ph.D.,[40] H. Zwenneke Flach, M.D.,[23] Henk A. Marquering Ph.D.,[3,39] Marieke E.S. Sprengers, M.D., Ph.D.,[3] Sjoerd

F.M. Jenniskens, M.D., Ph.D.,[27] Ludo F.M. Beenen, M.D.,[3] René van den Berg, M.D., Ph.D.,[3] Peter J. Koudstaal, M.D., Ph.D.,[1] Wim H. van Zwam, M.D., Ph.D.,[#,6] Yvo B.W.E.M. Roos, M.D., Ph.D.,[#,4] Aad van der Lugt, M.D., Ph.D.,[#,2] Robert J. van Oostenbrugge, M.D., Ph.D.,[#,5] Charles B.L.M. Majoie, M.D., Ph.D.,[#,3] and Diederik W.J. Dippel, M.D., Ph.D.[#,1]
* Berkhemer, Fransen and Beumer contributed equally.
# van Zwam, Roos, van der Lugt, van Oostenbrugge, Majoie and Dippel contributed equally.

Data monitoring and safety board

Chair: Martin M. Brown, National Hospital for Neurology & Neurosurgery, London, UK. Member: Thomas Liebig, Med. Fakultat, Univ Koln, Germany, Independent Statistician: Theo Stijnen, Leiden University Medical Center, Leiden, the Netherlands

Advisory board

Tommy Andersson, neuro interventionist, Karolinska Univeristy Hospital, Stockholm, Sweden, Heinrich Mattle, neurologist, University hospital, Bern, Switzerland, Nils Wahlgren, neurologist, Karolinska Hospital, Stockholm, Sweden.

Research nurses / local trial coordinators

Esther van der Heijden, Naziha Ghannouti; Erasmus MC University Medical Center Rotterdam, the Netherlands. Nadine Fleitour, Imke Hooijenga; Academic Medical Center Amsterdam, the Netherlands. Corina Puppels, Wilma Pellikaan; Sint Antonius Hospital, Nieuwegein, the Netherlands. Annet Geerling; Radboud University Nijmegen Medical Center, the Netherlands. Annemieke Lindl-Velema; Maastricht University Medical Center, the Netherlands. Gina van Vemde; Isala Klinieken, Zwolle, The Netherlands. Ans de Ridder, Paut Greebe, University Medical Center Utrecht, the Netherlands. Jose de Bont-Stikkelbroeck, Sint Elisabeth Hospital, Tilburg, the Netherlands. Joke de Meris, MC Haaglanden, the Hague, the Netherlands. Kirsten Janssen, Leiden University Medical Center, the Netherlands. Willy Struijk, HAGA Hospital, the Hague, the Netherlands.

PhD / Medical students

Silvan Licher, Nikki Boodt, Adriaan Ros, Esmee Venema, Ilse Slokkers, Raymie-Jayce Ganpat, Maxim Mulder, Nawid Saiedie, Alis Heshmatollah, Stefanie Schipperen, Stefan Vinken, Tiemen van Boxtel, Jeroen Koets; Erasmus MC University Medical Center Rotterdam, the Netherlands. Merel Boers, Emilie Santos, Jordi Borst, Ivo Jansen, Manon Kappelhof, Marit Lucas, Ralph
Geuskens, Renan Sales Barros, Roeland Dobbe, Marloes Csizmadia; Academic Medical Center Amsterdam, the Netherlands.

List of affiliations

Department of Neurology[1], Radiology[2], Public Health[40], Erasmus MC University Medical Center;
Department of Radiology and Nuclear Medicine[3], Neurology[4], Biomedical Engineering & Physics[39], Amsterdam UMC, University of Amsterdam, Amsterdam;

Department of Neurology[5], Radiology[6], Maastricht University Medical Center and Cardiovascular Research Institute Maastricht (CARIM);
Department of Neurology[7], Radiology[8], Sint Antonius Hospital, Nieuwegein;
Department of Neurology[9], Radiology[10], Leiden University Medical Center;
Department of Neurology[11], Radiology[12], Rijnstate Hospital, Arnhem;
Department of Radiology[13], Neurology[14], Haaglanden MC, the Hague;
Department of Neurology[15], Radiology[16], HAGA Hospital, the Hague;
Department of Neurology[17], Radiology[18], University Medical Center Utrecht;
Department of Neurology[19], Neurosurgery[20], Radiology[27], Radboud University Medical Center, Nijmegen;
Department of Neurology[21], Radiology[26], Elisabeth-TweeSteden ziekenhuis, Tilburg;
Department of Neurology[22], Radiology[23], Isala Klinieken, Zwolle;
Department of Neurology[24], Radiology[25], Reinier de Graaf Gasthuis, Delft;
Department of Neurology[28], Radiology[29], University Medical Center Groningen;
Department of Neurology[30], Radiology[31], Atrium Medical Center, Heerlen;
Department of Neurology[32], Radiology[33], Catharina Hospital, Eindhoven;
Department of Neurology[34], Radiology[35], Medisch Spectrum Twente, Enschede;
Department of Radiology[36], Neurology[42], Amsterdam UMC, Vrije Universiteit van Amsterdam, Amsterdam;
Department of Radiology[37], Noordwest Ziekenhuisgroep, Alkmaar;
Department of Radiology[38], Texas Stroke Institute, Texas, United States of America.
Department of Radiology[41], Massachusetts General Hospital, Boston, United States of America;
Department of Neurology[43], Radiology[44], Sint Lucas Andreas Hospital, Amsterdam, the Netherlands;
